# BioMaster: An Integrated Database and Analytic Platform to Provide Comprehensive Information About BioBrick Parts

**DOI:** 10.3389/fmicb.2021.593979

**Published:** 2021-01-21

**Authors:** Beibei Wang, Huayi Yang, Jianan Sun, Chuhao Dou, Jian Huang, Feng-Biao Guo

**Affiliations:** ^1^School of Life Science and Technology, University of Electronic Science and Technology of China, Chengdu, China; ^2^Centre for Informational Biology, University of Electronic Science and Technology of China, Chengdu, China

**Keywords:** synthetic biology, genetic circuit design, database integration, BioBrick parts, iGEM Registry

## Abstract

Synthetic biology seeks to create new biological parts, devices, and systems, and to reconfigure existing natural biological systems for custom-designed purposes. The standardized BioBrick parts are the foundation of synthetic biology. The incomplete and flawed metadata of BioBrick parts, however, are a major obstacle for designing genetic circuit easily, quickly, and accurately. Here, a database termed BioMaster http://www.biomaster-uestc.cn was developed to extensively complement information about BioBrick parts, which includes 47,934 items of BioBrick parts from the international Genetically Engineered Machine (iGEM) Registry with more comprehensive information integrated from 10 databases, providing corresponding information about functions, activities, interactions, and related literature. Moreover, BioMaster is also a user-friendly platform for retrieval and analyses of relevant information on BioBrick parts.

## Introduction

As a branch of the emerging biological sciences in the 21st century, synthetic biology is dedicated to design and construct novel biological parts, devices, and systems for specific purposes, and to make the process of engineering biology easier (Heinemann and Panke, 2006; Bartley et al., 2017). One of the important directions for synthetic biology is to build standard systems using standard devices which are comprised of standard parts. Therefore, to standardize biological parts and devices, Tom Knight at MIT introduced the concept of BioBrick standard in 2003 (Smolke, 2009).

BioBrick parts are biological parts that conform to a restriction-enzyme-based assembly standard, containing defined prefix and suffix sequences which can be recognized by specific restriction endonucleases (Canton et al., 2008; Shetty et al., 2008). Different assembly methods are constantly being reported, such as 3A assembly (Shetty et al., 2008), Gibson assembly (Gibson et al., 2009), Golden Gate assembly (Engler et al., 2008), MIDAS (van Dolleweerd et al., 2018), and CasHRA (Zhou et al., 2016). The standardization of BioBrick parts makes the compatibility and assembly of different biological parts more efficient, and the development of tools for biological computer-aided design (CAD) possible. Since the first successfully synthetic toggle switch and repressilator (Gardner et al., 2000), several related CAD tools, aiming at making the design easier and high throughput, have been developed, such as GenoCAD (Cai et al., 2010), TinkerCell (Chandran et al., 2009), Gene Designer (Villalobos et al., 2006), and Cello (Nielsen et al., 2016; Chen et al., 2020). GenoCAD is a computer tool that helps users assemble genetic circuits from a rich library of parts, compliantly with any of six implemented BioBrick standards. TinkerCell is a visual modeling tool that supports a hierarchy of biological parts. Gene Designer is a software for fast and easy design of synthetic DNA segments and uses advanced algorithms for codon optimization. And Cello provides a design environment for genetic circuit design automation. Thus, engineering biology, both manually and with CAD platforms, requires large amounts of BioBrick data of high quality (Nora et al., 2019). However, these powerful design processes are not supported by adequate information of standardized biological parts (Decoene et al., 2018).

So far, several data registries and repositories have been established to collect biological parts. For example, the Virtual Parts Repository consists of about 3000 biological parts (Cooling et al., 2010; Misirli et al., 2014), PAMDB contains 118 circuits and 165 parts (Huynh and Tagkopoulos, 2016), JBEI-ICE includes about 2302 items (Ham et al., 2012), SEVA-DB has a number of 185 vectors (Martinez-Garcia et al., 2015), while the international Genetically Engineered Machine (iGEM) Registry of standard biological parts (Smolke, 2009; Vilanova and Porcar, 2014) is, currently, the largest BioBrick database, containing over 40,000 items, cataloged as promoters, ribosomal binding sites (RBS), coding sequences, terminators, and so on. However, the iGEM Registry lacks joint analysis and retrieval with other databases, which are numerous and contain various biochemical, genetic, and molecular biological information.

Therefore, in this study, to complement and improve the metadata of iGEM BioBrick parts, we integrated 11 traditional biological databases (Supplementary Table S1), including iGEM Registry, UniProt (Bateman et al., 2017), QuickGO (Binns et al., 2009), KEGG (Kanehisa et al., 2017), BioGRID (Oughtred et al., 2019), BRENDA (Jeske et al., 2019), ExplorEnz (McDonald et al., 2009), STRING (Szklarczyk et al., 2017), PubMed (Sayers et al., 2020), EPD (Perier et al., 2000), and PromEC (Hershberg et al., 2001). These databases supplement the information of functions, sites, interactions, feature keys, and references. The integrated database, termed BioMaster, also provides a more user-friendly platform for searching, browsing, and analyzing BioBrick information.

## Materials and Methods

**FIGURE 1:**
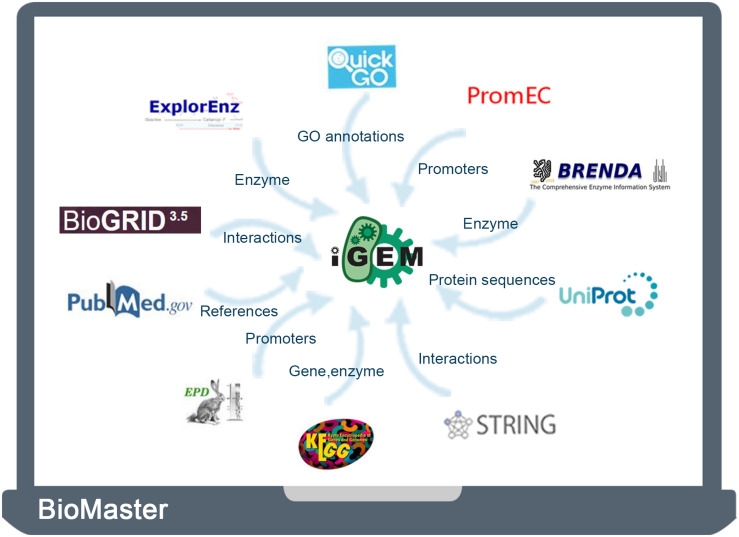
Strategy of data acquisition and integration.

In this study, the UniProt database was taken as a hub for connecting the iGEM Registry with other databases. We first downloaded the entire BioBrick database from the iGEM Registry^1^. The database contains the parts table, the sequence, and features of BioBrick parts (O’Donovan et al., 2002). Then BLAST was used to find matched sequence in the UniProt database. The other nine databases were then integrated through UniProt unique identifier. Finally, we developed a user-friendly database, termed BioMaster, for easy searching, browsing, and analyzing all these BioBrick information (Figure 1).

BioMaster^2^ was developed with PHP, HTML, CSS, JavaScript, and Python on a Linux platform. Laravel (version7.0.0^3^), a PHP web framework, was used in the back end. Bootstrap^4^ and Vue^5^ were mainly used to build interactive pages of the front end of the website. The data were stored in a MySQL database and can be automatically updated. We also packed all data, programs, and the operating environment, and uploaded to the docker hub^6^. Elastic search^7^, a safe, efficient, and well-structured search engine, was used to implement full-text search, fuzzy search, and keyword search.

### Data Sorting of the Sequence Alignment Results

There was no information associated with other databases in the iGEM Registry. To get the corresponding information, UniProt was used as a hub for connecting the iGEM Registry with other nine databases. The connection between the iGEM Registry and UniProt was established by sequence alignment on featured (functional) sequences in iGEM Registry or full BioBrick sequences in case of no available feature information. The sequence alignment was performed by BLAST (Altschul et al., 1997). Each alignment result of BLAST includes three main parameters: Score, expect value (E-value), and Identity. The Score is the sum of similarity of each pair between two sequence, so it is related to the length of the sequence. The E-value is the expected number of high score pairs and decreases exponentially as the score increases. The Identity is the proportion of which two sequences have the same residues at the same position after alignment. The results of BLAST are always displayed with a descending Score. In most cases, however, the order is different if a different standard is used for sorting. Therefore, to comprehensively consider all three parameters, we used their weighted average (*p*), calculated as the following:

$$\begin{array}{c} p=Identity\times w1+\frac{Score}{S_{max}\times length}\\ \times w2+\frac{log_{6}\left(-lg\left(Evalue\right)\right)}{3}\times w3, \end{array}$$

where *w*1, *w*2, and *w*3 are their weights, respectively. As the Identity is ranged from 0 to 1, the Score and E-value were adjusted to this range. The Score was divided by the maximum in the substitution matrix (*S*_*max*_) (Altschul, 1991) and the length of the query sequence. After taking the negative logarithm of 10 of E-value, the value falls into a range of 1–200. To adjust it to 0–1, we calculated the logarithm of 6 again and divide it by 3 as shown in the above equation.

To determine the value of *w*1, *w*2, and *w*3, we tested from 0.1 to 0.8 with an interval of 0.1, respectively. About 200 items of BLAST results, manually selected, formed a training set, to obtain optimal combination. We found that the screening accuracy was highest when *w*1 = 0.1, *w*2 = 0.5, and *w*3 = 0.4.

To validate the data sorting model, we labeled about 8500 BLAST results, which were classified by our model and support vector machine (SVM). The threefold validation accuracy of SVM is around 0.93, lower than the accuracy of our model (about 0.98). The area under ROC curve (AUC) value of our model is 0.99, which is also better than the AUC value of SVM (0.94) (Supplementary Figure S1A). Meanwhile, the average of Score, E-value, and Identity of the positive items also demonstrates that the performance of our model is better than SVM (Supplementary Figure S1B).

## Results and Web Interface

To complete the information of iGEM BioBrick parts, we developed a database termed BioMaster, which provides a user-friendly query interface and a set of tools for a comprehensive overview of BioBrick parts.

### The Amount of Data

BioMaster has all the data from the iGEM Registry. In addition, it also integrates corresponding information from 10 databases (UniProt, STRING, BioGRID, KEGG, QuickGO, PubMed, BRENDA, ExplorEnz, EPD, and PromEC) to complement the iGEM BioBrick datasheet. The iGEM Registry contains 47,934 entries of BioBrick information, among which 44,565 entries have feature information. Through sequence alignment with UniProt database and data screening, we found corresponding 3747 items in UniProt. Via their UniProt IDs, information from other nine databases was integrated: 100,000 items of interaction information from STRING and BioGRID, 94,260 pieces of annotations from QuickGO, 6203 pieces of gene information from KEGG, 2694 pieces of homology information from KO, information and annotations of 1336 enzymes from KEGG ENZEMY, BRENDA, and ExplorEnz, some promoters information from EPD and PromEC, and more than 1 million associated references from PubMed. According to the iGEM Registry, BioBrick parts are divided into 25 categories (Supplementary Table S2), of which the eight main categories are promoters, RBS, coding sequences, translational units, terminators, DNA, plasmids, and composite parts.

### Search Module

**FIGURE 2:**
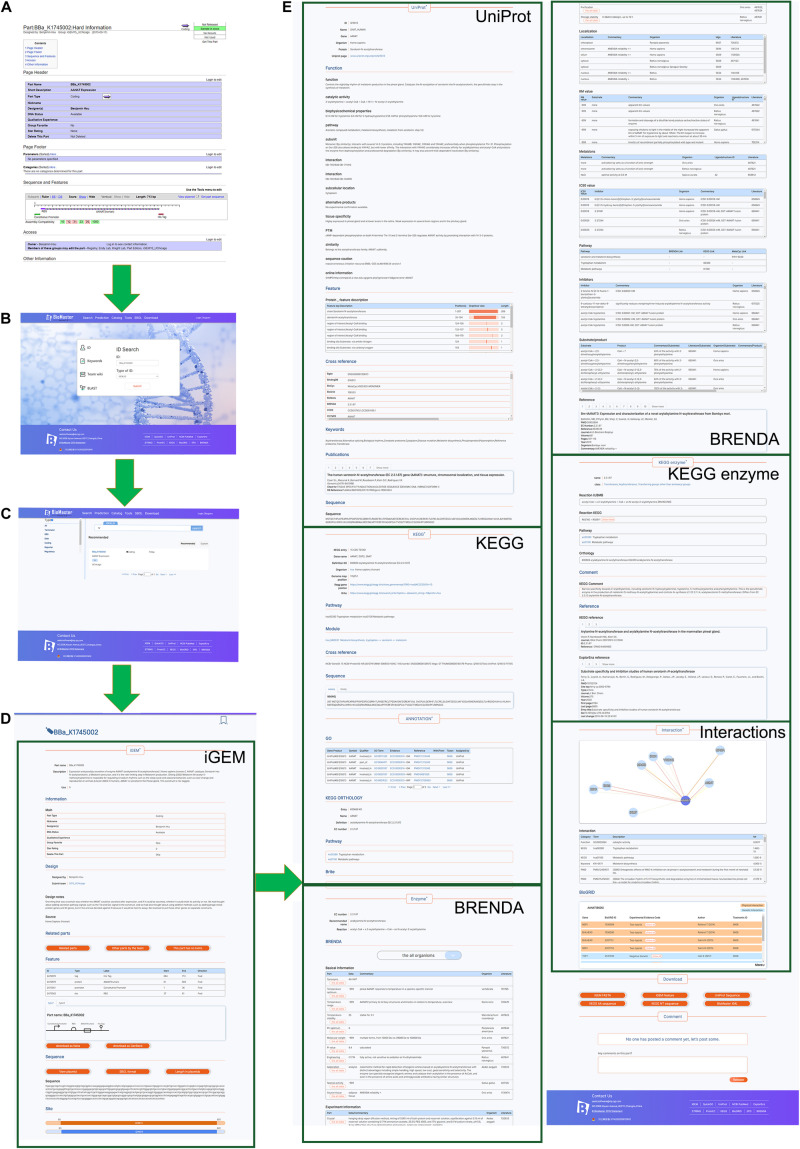
Snapshots of BioMaster. **(A)** The data in iGEM Registry, taking the BioBrick BBa_K1745002 as an example. **(B)** The searching page of BioMaster. **(C)** Search result page of BBa_K1745002. **(D,E)** Information of BBa_K1745002 in BioMaster database. For detailed contents, please check on the website.

BioMaster supports a total of four search methods: ID search, Keyword search, Team Wiki search, and sequence alignment by BLAST (Figure 2B). The ID search automatically recognizes IGEM_ID, EPD_ID, UniProt_ID, and Gene_Name. Keywords search returns parts in the iGEM Registry related to the keywords. Team Wiki can search for the team names and keywords related to the iGEM teams. Searching for an amino acid sequence or nucleotide sequence is also practicable via BLAST against BioMaster database.

The search results include a total of no more than 1000 related items sorted into different groups (Figure 2C), such as terminators, DNA, RNA, and reporters. The detailed information can be accessed by selecting any item of results. It shows the information obtained from the iGEM Registry (Figure 2D) as well as other databases related to the part in detail (Figure 2E). In order to display the interaction between different parts or parts and proteins clearly, cytoscape.js was implemented to show interaction diagrams. The information of the corresponding BioBrick parts will be shown by clicking on the nodes and links. In addition, synthetic biology open language (SBOL) is used to visualize the feature information and display it as a graph. The search results can be freely downloaded and is open for user’s comments. Detailed usage tutorials are available on our website in the form of videos^8^.

### Application

Taking the part BBa_K1745002 for example, Figure 2A shows all the information for this part in the iGEM Registry, from which we found that this part is an enzyme, aralkylamine N-acetyltransferase (AANAT), but there is almost no information other than the sequence and features. In our database, BBa_K1745002 was matched to the UniProt_ID Q16613 correctly (Figure 2E), which provides the information about its function, catalytic activity, structure, related reference, and so on. Via the UniProt_ID, massive information, such as related pathways, optimal experimental conditions, KM values, IC50 values, inhibitors, and interaction networks, was integrated from KEGG, Brenda, String, and BioGrid. Therefore, this part is better characterized in our database.

### Tools for Analyses and Visualization of BioBrick Parts

**FIGURE 3:**
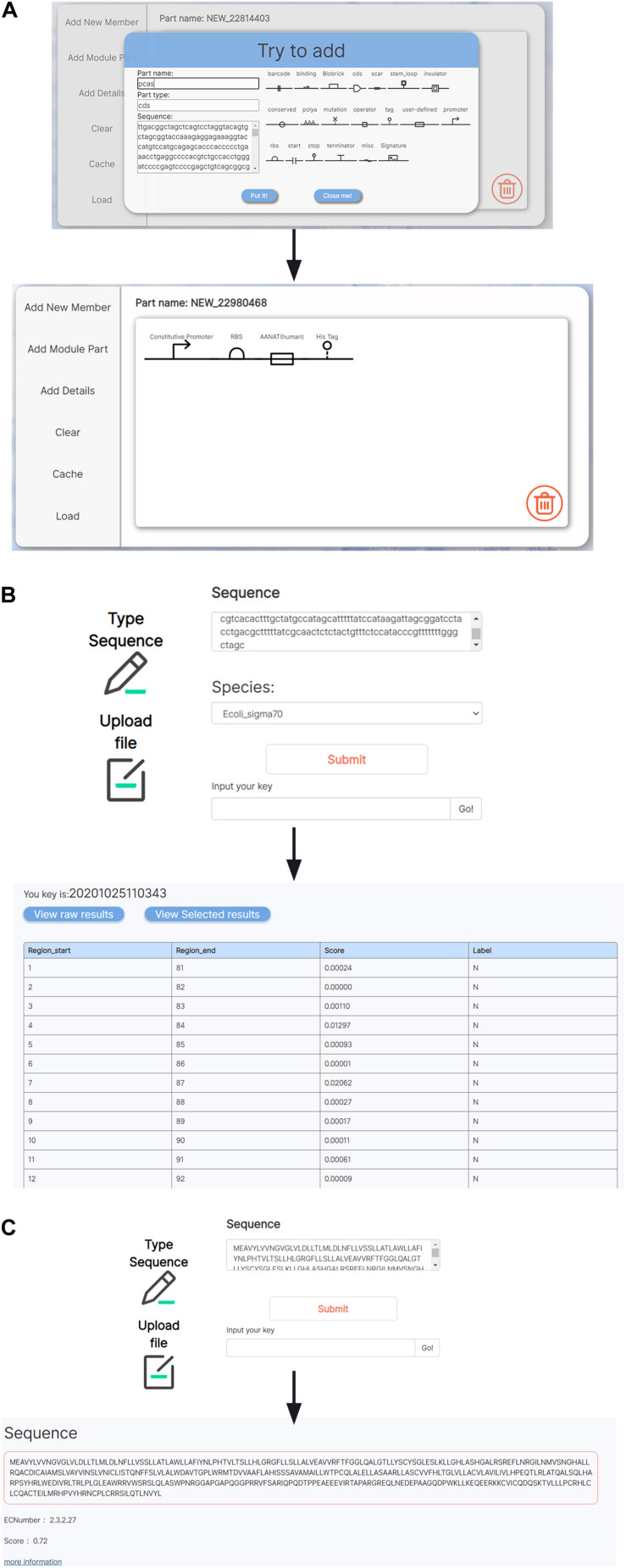
Tools for BioBrick analyses and visualization. **(A)** A SBOL-based visualization tool for genetic circuit design. **(B)** Promoter prediction. **(C)** EC number prediction.

Several tools for analyses and visualization of BioBrick parts were integrated in BioMaster. We integrated a JAVA-based visual aiding design software, SBOL designer, into BioMaster to provide visualization and designing of biological components (Figure 3A) (Roehner et al., 2016; Madsen et al., 2019). On the other hand, concerning the limit amount of BioBrick entries, BioMaster provides two prediction tools to conduct bioinformatic exploration on unknown sequences: the promoter prediction (Umarov and Solovyev, 2017) and the enzyme commission (EC) number prediction (Dalkiran et al., 2018). The promoter prediction implemented uses convolutional neural network to recognize both prokaryotic and eukaryotic promoters (Figure 3B). The EC prediction was integrated into BioMaster to predict the enzymatic function for uncharacterized protein sequence. An EC number is given as the prediction result, and more information can be obtained in BRENDA via the EC number (Figure 3C).

## Summary

The design of genetic circuit requires reliable, comprehensive, and complete information of BioBrick parts. To complete the BioBrick metadata of the iGEM Registry, we developed a database called BioMaster. Various information from 10 popular databases was integrated to the iGEM parts with diverse and detailed information. Meanwhile, BioMaster provides convenient retrieval, user-friendly interface, and potential predictions. In the future, we will integrate more data in breadth and depth from other synthetic biological databases and publications to connect the massive amounts of data pieces (Nora et al., 2019), and further improve the analysis tools on BioMaster. It is hoped that BioMaster could provide a platform to connect and standardize scattered biological part data, and to make designing of new biological systems easier both automatically and manually.

## Data Availability Statement

Publicly available datasets were analyzed in this study. These data can be found here: http://www.biomaster-uestc.cn.

## Author Contributions

HY, JS, and CD collected the data, analyzed the data, developed the web, and wrote the manuscript. JH and F-BG designed the database and wrote the manuscript. BW designed the database, analyzed the data, and wrote the manuscript. All authors contributed to the article and approved the submitted version.

## Conflict of Interest

The authors declare that the research was conducted in the absence of any commercial or financial relationships that could be construed as a potential conflict of interest.

## Footnotes

1 http://parts.igem.org/Registry_API

2 http://www.biomaster-uestc.cn

3 https://laravel.com/

4 https://v2.bootcss.com/

5 https://cn.vuejs.org/

6 https://hub.docker.com/u/igem2019uestcsoftware

7 https://www.elastic.co/cn/

8 http://www.biomaster-uestc.cn/public/index.php/main/tutorial
