# Supplementary material for: BioMaster: An Integrated Database and Analytic Platform to Provide Comprehensive Information About BioBrick Parts
Source: Front Microbiol. 2021 Jan 21;12:593979. doi: 10.3389/fmicb.2021.593979 (PMC7858672; doi:10.3389/fmicb.2021.593979)
Supplement: Supplementary file 1 [file Data_Sheet_1.PDF]

Supporting information

BioMaster: an integrated database and analytic platform to provide comprehensive information about BioBrick parts

Huayi Yang[a, ‖], Jianan Sun[a, ‖], Chuhao Dou[a, ‖], Jian Huang[a,b], Fengbiao Guo[a,b], and Beibei Wang[a,b,*]

[a]School of Life Science and Technology, University of Electronic Science and Technology of China, 2006 Xiyuan Ave, Chengdu, Sichuan, 611731, China.

[b]Center for Informational Biology, University of Electronic Science and Technology of China, 2006 Xiyuan Ave, Chengdu, Sichuan, 611731, China.

[‖]They contributed equally to this work.

* Corresponding Author, bbwang@uestc.edu.cn.

Table S1. The introduction of databases integrated in Biomaster.

Name	Description	URL
iGEM Registry	A collection of BioBrick parts that are mostly submitted by the iGEM teams.	http://parts.igem.org/Main_Page
UniProt	A large resource of protein sequences and associated detailed annotation.	https://www.**uniprot**.org
QuickGO	A fast web-based browser of the Gene Ontology and Gene Ontology annotation data.	https://www.ebi.ac.uk/QuickGO/
KEGG	A database resource for understanding high-level functions and utilities of the biological system, such as the cell, the organism and the ecosystem, from genomic and molecular-level information.	https://www.kegg.jp/
BioGRID	A curated biological database of protein-protein interactions, genetic interactions, chemical interactions, and post-translational modifications.	https://thebiogrid.org/
BRENDA	An enzyme database containing experiment information, localization, KM value, pathway, IC50 value, inhibitors and so on.	https://www.brenda-enzymes.org/
ExplorEnz	A database that is used for the curation and dissemination of the IUBMB Enzyme Nomenclature.	https://www.enzyme-database.org/class.php
STRING	A database of known and predicted protein–protein interactions.	https://string-db.org/
PubMed	A free search engine accessing primarily the MEDLINE database of references and abstracts on life sciences and biomedical topics.	https://www.nlm.nih.gov/bsd/pubmed.html
EPD	A biological database and web resource of eukaryotic RNA polymerase II promoters with experimentally defined transcription start sites.	https://epd.epfl.ch//
PromEC	An updated compilation of Escherichia coli mRNA promoter sequences.	http://margalit.huji.ac.il/promec/

Table S2. The number of BioBrick parts of different categories in iGEM Registry and BioMaster.

Part type	Description	Count
Promoters	Promoters are DNA sequences that tend to recruit transcription mechanisms and cause transcription of downstream DNA sequences	4167
Ribosome binding site	A ribosome binding site (RBS) is an RNA sequence found in mRNA to which ribosomes can bind and initiate translation.	769
Protein domains	Protein domains are portions of proteins cloned in frame with other proteins domains to make up a protein coding sequence.	1301
Protein coding sequences	Protein coding sequences are DNA sequences which encode the amino acid sequence of a particular protein.	10257
Translational units	A translational unit comprise a ribosome binding site and a protein coding sequence.	880
Terminators	A terminator is an RNA sequence that usually occurs at the end of a gene or operon mRNA and causes transcription to stop.	518
DNA	DNA parts provide functionality to the DNA itself. DNA parts include cloning sites, scars, primer binding sites, spacers, recombination sites, conjugative tranfer elements, transposons, origami, and aptamers.	1717
Plasmid backbones	A plasmid backbone is defined as the plasmid sequence beginning with the BioBrick suffix, including the replication origin and antibiotic resistance marker, and ending with the BioBrick prefix.	454
Plasmids	A plasmid is a circular, double-stranded DNA molecules typically containing a few thousand base pairs that replicate within the cell independently of the chromosomal DNA.	674
Primers	A primer is a short single-stranded nucleic acid utilized by all living organisms in the initiation of DNA synthesis.	685
Composite parts	Composite parts are combinations of of two or more BioBrick parts.	9961
Reporter	All of the reporter genes encode for fluorescent proteins (FP's).	2360
RNA	These parts code for RNA segments with novel structures or functions, such as stem-loop riboregulators. All of these contain promoter regions regulating RNA production.	976
Project	A complete part that can achieve certain functions.	656
Generator	A composite-type part based on the combination of a Protein Coding region with one or more other parts. Protein Generators are devices that enable expression of the mRNA encoded by the CDS.	2425
Inverter	Classically, a genetic inverter receives as input the concentration of repressor A and, via gene expression, sends as output the concentration of repressor B.	116
Intermediate	Intermediates for building complex systems.	3506
Signalling	signalling devices allow communication between an individual cell and its neighbors in culture or on a plate.	511

Measurement	These systems allow measurement of the relative strength of some types of basic parts.	1162
Temporary	Temporary parts, most of which are used for testing.	866
Other	Other unclear parts.	1419
Cell	The parts in the Registry operate in Escherichia coli chassis,Bacillus subtilis chassis, etc.	75
Device	A device is a type of composite part that conducts an operation in the cell.	2277
Conjugation	The conjugative plasmid, which transferring genetic information between two bacterial cell containing OriT and TraJ.	51
T7	DNA sequence constructed with T7 phage gene as template.	57
Scar	A scar is a DNA sequence that is a byproduct of assembling samples of DNA parts together.	121

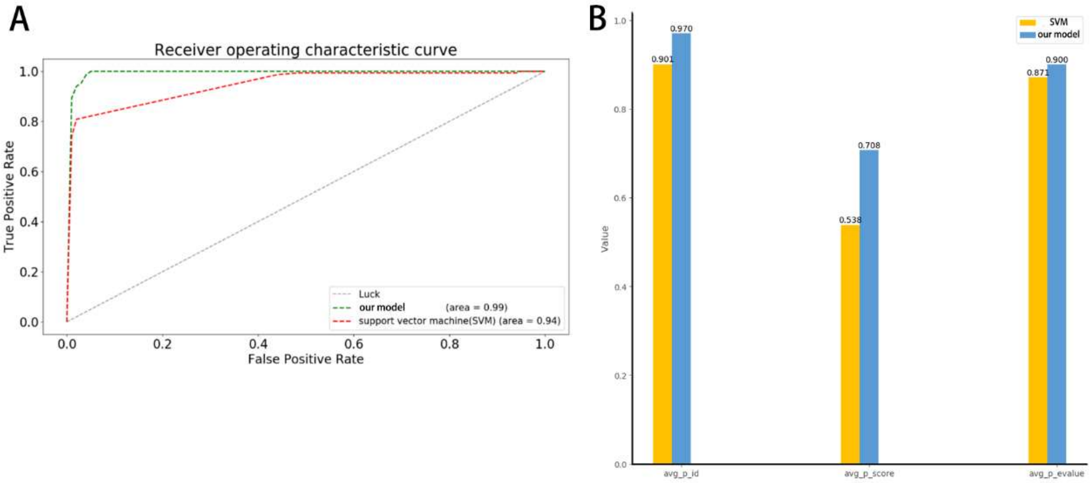

Figure S1. (A) ROC curves of our model (green) and SVM (red). (B) The average values of Score, E-value and Identity of the positive items of our model (blue) and SVM (yellow).
